# Differential neurodegenerative phenotypes are associated with heterogeneous voiding dysfunction in a coronavirus-induced model of multiple sclerosis

**DOI:** 10.1038/s41598-019-47407-x

**Published:** 2019-07-26

**Authors:** Sanghee Lee, Balachandar Nedumaran, Joseph Hypolite, Brian Caldwell, Michael C. Rudolph, Anna P. Malykhina

**Affiliations:** 1 Department of Urology, University of California San Diego, La Jolla, California USA; 2 Division of Urology, Department of Surgery, University of Colorado Denver, Aurora, Co USA; 3 Division of Endocrinology, Metabolism & Diabetes, University of Colorado Denver, Aurora, Co USA; 4 NORC Metabolic and Cellular Analysis Core Center for Women’s Health Research, University of Colorado Denver, Aurora, Co USA

**Keywords:** Neurological disorders, Bladder

## Abstract

Patients with multiple sclerosis (MS) develop a variety of lower urinary tract symptoms (LUTS). We previously characterized a murine model of neurogenic bladder dysfunction induced by a neurotropic strain of a coronavirus. In the present study, we further study the role of long-lasting neurodegeneration on the development of neurogenic bladder dysfunction in mice with corona-virus induced encephalitis (CIE). Long-term follow up study revealed three phenotypes of neurodegenerative symptom development: recovery (REC group), chronic progression (C-PRO group) and chronic disease with relapsing-remitting episodes (C-RELAP group). The levels of IL-1β in REC group, IL-10 in C-RELAP group, and IL-1β, IL-6, IL-10 and TNF-α in C-PRO group were diminished in the brain. The levels of TNF-α in REC group and INF-γ, IL-2, TGF-β and TNF-α in the C-PRO group were also diminished in the urinary bladder. Mice in C-RELAP group showed a delayed recovery of voiding function. *In vitro* contractility studies determined a decreased basal detrusor tone and reduced amplitude of nerve-mediated contractions in C-RELAP group, whereas C-PRO group had elevated muscle-mediated contractions. In conclusion, mice with CIE developed three phenotypes of neurologic impairment mimicking different types of MS progression in humans and showed differential mechanisms driving neurogenic bladder dysfunction.

## Introduction

Neurogenic bladder dysfunction often develops in patients with neurodegenerative disorders such as multiple sclerosis (MS) causing a great discomfort, and having a negative effect on the quality of the individual’s social, occupational and sexual life^1^. Up to 90% of patients with MS experience lower urinary tract symptoms (LUTS) including detrusor overactivity, urgency, frequency of urination, nocturia and dysuria. Prevalence and severity of LUTS increase with disease duration and progression of physical disability in MS patients^2^. In 10–15% of patients, bladder symptoms are present at the onset of MS when there may be few demyelinated lesions in the spinal cord and/or brain detected by the MRI^3^. The most common symptom reported by patients in remission is urgency of micturition followed by urinary frequency^3–6^. Bladder symptoms become prevalent^7^ in patients with chronic progression of the disease, and are often accompanied by bowel and sexual dysfunction^8,9^. Poor understanding of the mechanisms underlying functional interplay between the lower urinary tract (LUT) and the nervous system warrants further translational and clinical research efforts in the field.

Experimental autoimmune encephalomyelitis (EAE) is one of the most studied animal models of MS which is induced by inoculation of rats or mice with antibodies against myelin binding protein (MBP), proteolipid protein (PLP) or myelin oligodendrocyte glycoprotein (MOG)^10–12^. After inoculation, animals develop pathological features of inflammation and demyelination in the spinal cord and brain, with infiltration of the spinal cord with inflammatory cells expressing pro-inflammatory cytokines^10–12^. EAE-driven demyelination is mostly mediated by CD4 T cells of a Th17 phenotype^13^, which is a population of immune cells known to be associated with more severe pathology of MS^14,15^. Several previous studies investigated micturition abnormalities in animals with EAE. In rats with MBP-induced EAE, both detrusor areflexia and detrusor hyperactivity were observed and correlated with spinal cord inflammation and hindlimb paralysis^16^. Using cystometric analysis, Mizusawa *et al*. showed that EAE caused detrusor areflexia in 52% of female rats in comparison to 12% which developed detrusor overactivity^16^. Another group established that the majority of female rats showed symptoms of detrusor areflexia prior to EAE onset but that detrusor overactivity developed at the onset of the disease^17^. Additional animal models of neurodegeneration and demyelination include a murine model of coronavirus-induced encephalomyelitis (CIE)^18,19^. Mice inoculated with neurotropic strain A59 of mouse hepatitis virus (MHV) develop CIE accompanied by severe central nervous system (CNS) demyelination and axonal degeneration^20,21^. The viral nature of CIE model reflects a spontaneously occurring demyelination in the CNS in comparison to induced models (*e.g*. EAE). MHV-driven demyelination is mediated by interferon-gamma (INF-γ) of a Th1 phenotype^18,19,22^. Compelling preponderance of Th1 cytokine responses has been previously reported in a majority of MS patients^15,23,24^.

We previously characterized the symptoms of neurogenic voiding dysfunction in CIE mice^21,25^. Several neural mechanisms contributing to neurogenic bladder in the CIE model of MS were identified in our laboratory. They included the morphological changes in the neuronal centers controlling micturition, activation of gliosis at the lesion sites in the spinal cord, increased expression of pro-inflammatory cytokines during acute inflammation in the CNS followed by an up-regulation of anti-inflammatory cytokines at later time points, and an increase of purinergic component of nerve-mediated bladder contractions associated with the changes in bladder mucosa^21,25^. Most of these changes were observed within a month after inoculation with the virus, when the first signs of demyelination developed in the CNS. In the present study, we aimed to understand the long-term effects of neurodegenerative changes on voiding patterns and bladder function in CIE model. We tested the hypothesis that long-lasting neurodegeneration in CIE mice may cause the development of differential neurological phenotypes of MS development associated with heterogeneous types of neurogenic LUTS.

## Results

### Long-term follow-up of CIE mice revealed three phenotypes of neurological symptom development

**Figure 1:**
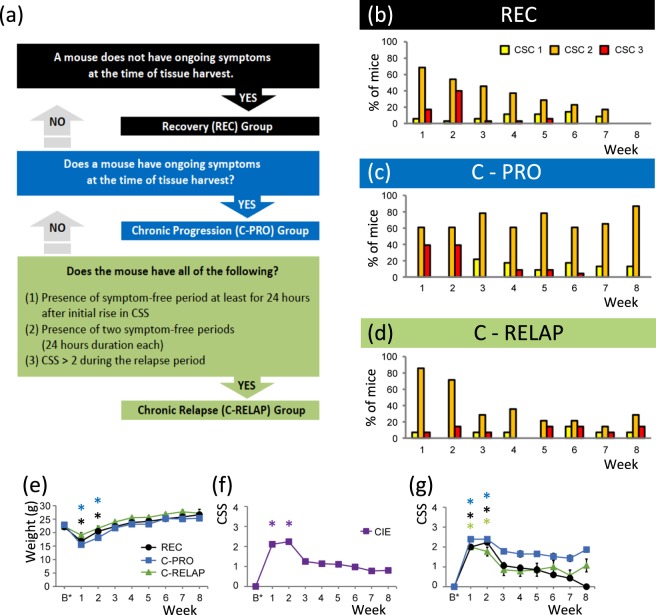
The long-term dynamic changes in neurological symptom development in CIE mice. (**a**) Algorithm for group assignment based on the severity and time-dependent changes in clinical symptom score (CSS). Fluctuations in CSS over a period of 8 weeks in the recovery (REC) group (**b**), chronic progression (C-PRO) group (**c**) and chronic progression group with relapsing-remitting episodes (C-RELAP) group (**d**). (**e**) Changes in body weight in all subgroups of CIE mice. (**f**) Time-dependent changes of CSS in all CIE mice. (**g**) Time-dependent changes in CSS in each of subgroups of CIE mice. *p ≤ 0.05 in comparison to baseline value, each color of *indicates comparison of CSS at each time point within the group (blue - C-PRO; black – REC; and green - C-RELAP groups).

To understand the effects of coronavirus-induced demyelination on LUT function, we performed a long-term follow up study (up to 10 wks) of CIE mice. As described in the Methods, all CIE mice were assigned to one of the following experimental groups: a group with recovery from initial symptoms after acute phase of the disease (REC group); a group with chronic progression of neurodegeneration and the presence of symptoms at 8 wks post-inoculation (C-PRO group), and a group with occurring relapsing and remission episodes (C-RELAP group, Fig. 1a).

Out of 72 mice in CIE group, 48.6% of animals (N = 35) had no neurological impairment symptoms at 8 wks and, therefore, were assigned to REC group (Fig. 1b). 31.94% of CIE mice (N = 23) still had elevated clinical symptom score (CSS) and were assigned to C-PRO group (Fig. 1c). 19.44% of all CIE mice (N = 14) experienced relapsing-remitting episodes and, therefore, were assigned to C-RELAP group (Fig. 1d). Inoculation of mice with MHV-A59 virus caused a significant weight loss at 1 and 2 wks in all groups of inoculated animals (Fig. 1e, p < 0.05 to baseline). The weight loss was reflective of the acute phase of infection associated with the development of active inflammation in the CNS. Mice in all groups started gaining weight at the normal rate at 3 wks post-inoculation.

The analysis of the combined CSS in all CIE mice showed that the most significant neurological impairment was developed between 1 and 2 weeks after inoculation with the virus (Fig. 1f, p < 0.05 to the baseline). The highest weekly recorded CSS revealed that up to 40% of mice in REC and C-PRO groups had CSS of 3 over the course of the experiment (Fig. 1g). Mice in C-PRO group showed the patterns of neurologic impairment similar to the mice in REC group at 1 and 2 wks, however, 86.96% and 13.04% of C-PRO mice still had CSS 2 and 1, respectively, at 8 wks post-inoculation (Fig. 1g). Interestingly, more mice in C-RELAP group (92.86% and 85.71% at 1 and 2 wks, respectively) had CSS 2 or above at earlier time points as compared to REC and C-PRO mice (Fig. 1g).

### Long-term follow-up of CIE mice revealed expression changes of cytokines and growth factor in the brain and urinary bladder

**Figure 2:**
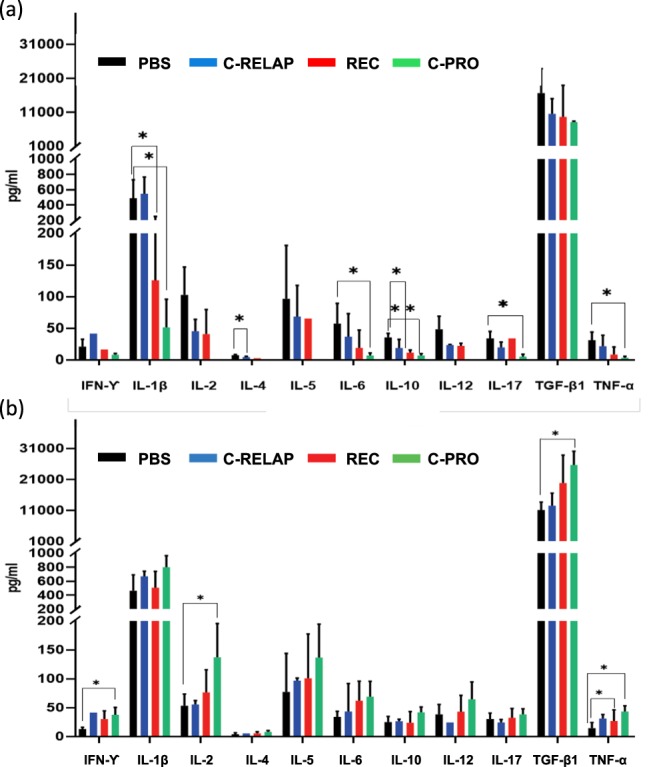
Protein expression profile of cytokines and growth factors. (**a**) Expression of INF-γ, IL-1β, IL-2, IL-4, IL-5, IL-6, IL-10, IL-12, IL-17, TGF-β and TNF-α in the brain of all subgroups of CIE mice utilizing ELISA multiplex assay after inoculation with the virus. *p ≤ 0.05 vs. control (PBS) group. (**b**) Expression of the cytokines and growth factors in the urinary bladder in the same groups of mice. *p ≤ 0.05 vs. control (PBS) group.

Our group previously reported that CIE mice had fluctuations in CNS cytokine levels at 4 wks post-inoculation^21^. Therefore, we also tested the expression level of INF-γ, IL-1β, IL-2, IL-4, IL-5, IL-6, IL-10, IL-12 and IL-17 as well as growth factors, TGF-β and TNF-α, by multiplex ELISA at 10 wks after inoculation with the virus. REC mice had significantly decreased expression levels of IL-1β and IL-10 in the brain (p < 0.05 to PBS group, Fig. 2a). C-PRO group was characterized by a decreased expression of IL-1β, IL-6, IL-10, IL-17 and TNF-α (p < 0.05 to PBS group, Fig. 2a) whereas C-RELAP mice had a significantly reduced level of IL-4 in the brain (p < 0.05 to PBS group). No significant changes in cytokine content were detected in the spinal cord of all subgroups of CIE mice (data not shown). However, several changes were noted in the urinary bladders of CIE mice. Figure 2b presents a significantly decreased expression of TNF-α in REC mice (p < 0.05 to PBS group) and a reduced content of INF-γ, IL-2, TGF-β and TNF-α in the urinary bladders of C-PRO mice (p < 0.05 to PBS group) without substantial changes observed in C-RELAP group (Fig. 2b).

### Evaluation of micturition patterns *in vivo* by FPA revealed a delayed recovery of voiding function in C-RELAP group

**Figure 3:**
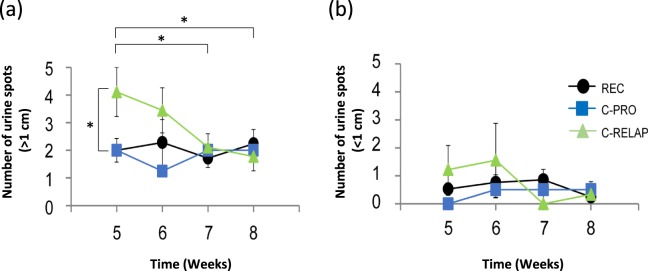
Micturition patterns in all subgroups of CIE mice evaluated by FPA after 5 weeks post-inoculation with the virus. (**a**) The number of large urine spots (≥1.0 cm) in REC, C-PRO and C-RELAP groups of CIE mice. (**b**) Analysis of small size urine spots (<1.0 cm) in the same groups of animals. *p ≤ 0.05 C-RELAP group vs REC group at 5 wks indicated by vertical line. *p ≤ 0.05 C-RELAP group at 5 wks vs at 7 and at 5 wks vs at 8 wks indicated by two horizontal lines.

We previously reported an increase in large (>1 cm) and small (<1 cm) voiding spots in CIE mice from 2 to 4 wks after inoculation with the same virus^21^ suggestive of overactive bladder phenotype development. In this study, we followed the micturition patterns up to 8 wks in each of REC, C-PRO and C-RELAP groups. The number of large urine spots in C-RELAP group was significantly higher than in REC group at 5 wks post-inoculation (Fig. 3a, *p* ≤ 0.05 to REC and C-PRO groups). In addition, the number of large urine spots in C-RELAP group at 5 wks was significantly higher than the number at 7 and 8 wks within the same group. No significant differences were observed in the numbers of small urine spots between the groups at long-term followup (Fig. 3b).

### Mice in C-RELAP group showed the most severe overactive bladder phenotype evaluated by cystometry

**Figure 4:**
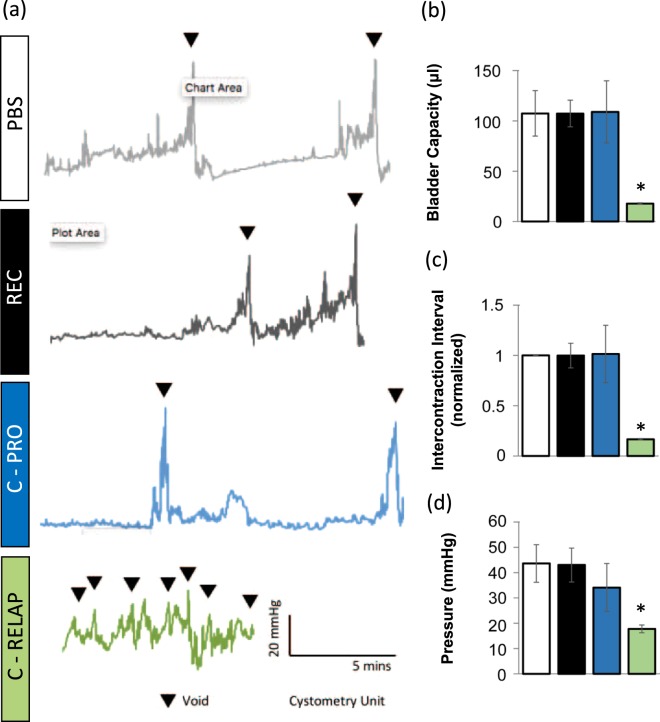
Cystometric evaluation of bladder function in control mice and in REC, C-PRO and C-RELAP groups of CIE mice. (**a**) Representative micturition cycles recorded in control and CIE subgroups. (**b**) Comparison of bladder capacity between control and experimental groups; (**c**) Inter-micturition interval normalized to the duration of the micturition cycle in control mice; (**d**) analysis of bladder pressure at micturition in REC, C-PRO and C-RELAP groups of CIE mice. *p ≤ 0.05 to respective control group (PBS) values.

While FPA assay allows for a long-term follow-up of micturition patters in mice *in vivo*, it does not provide the direct measures of bladder pressure, bladder capacity and frequency of micturition. Therefore, we performed urodynamic evaluation of voiding patterns in each subgroup of CIE mice by *in vivo* cystometry which was conducted in unanesthetized mice. Figure 4a shows representative single micturition cycles recorded in the control (PBS), REC, C-PRO and C-RELAP groups of mice. At 10 wks post-inoculation, bladder capacity (Fig. 4b), the inter-micturition interval (Fig. 4c), and bladder pressure at voiding (Fig. 4d) in all groups, except for the C-RELAP group, were similar to the respective values in the control group. However, mice in C-RELAP group developed overactive bladder phenotype associated with lower bladder capacity (17.88 ± 0.72 vs 107.49 ± 22.56 μl, p ≤ 0.05 to control group, Fig. 4b), reduced ratio of normalized inter-micturition interval (0.17 ± 0.01, *p* ≤ 0.05 ratio to control group, Fig. 4c), and decreased pressure at micturition (17.75 ± 1.52 vs. 43.62 ± 7.43 ml, *p* ≤ 0.05 to control; Fig. 4d). These results suggest that mice in C-RELAP group develop a more severe and long-lasting type of neurogenic bladder overactivity than other groups providing evidence of some correlation between the type of neurodegenerative changes in the CNS and type of developed voiding dysfunction in CIE mice.

### *In vitro* studies revealed differential contractile responses of the detrusor to electric field stimulation (EFS) among the CIE groups

**Figure 5:**
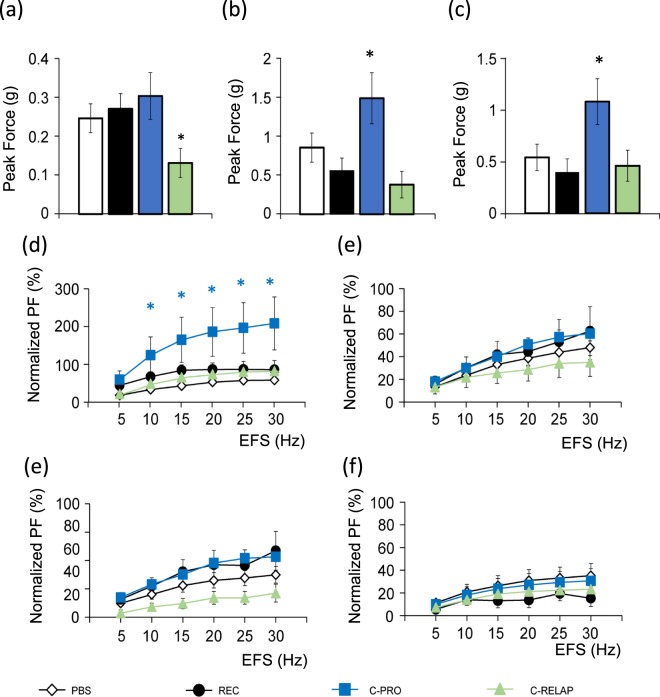
Contractile responses of bladder smooth muscle (BSM) strips in response to different stimulation. (**a**) Muscle tone at baseline in REC, C-PRO and C-RELAP groups of CIE mice in response to electric field stimulation (EFS). (**b**) Maximal muscle strength (L0), (**c**) amplitude of contractions (Peak Force, g) in response to 125 mM KCl. (**d**) Purinergic component of the contractile response of BSM to electrical field stimulation (EFS). (**e**) Cholinergic component of BSM contractile response to EFS. (**f**) Muscarinic component of BSM contractile response to EFS. (**g**) Nicotinic component of BSM contractile response to EFS. *p ≤ 0.05 to respective control group (PBS) values.

**Figure 6:**
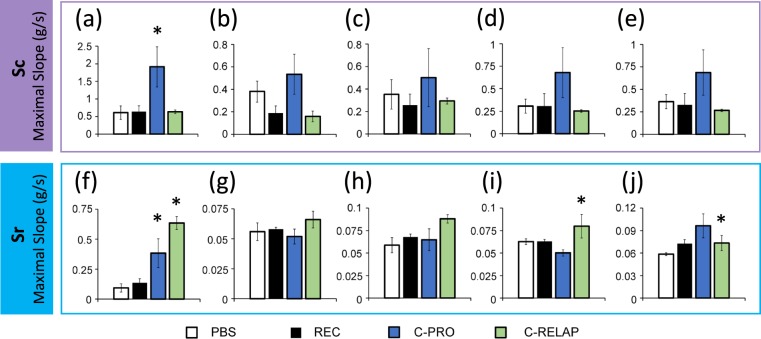
The slope values of contraction (Sc) and relaxation (Sr) phases of bladder smooth muscle (BSM) contractile response after EFS. (**a**) Sc of purinergic-mediated response. (**b**) Sc of M3-dependent muscarinic response. (**c**) Sc of M2-dependent muscarinic response. (**d**) Sc of α3-receptor mediated nicotinic response. (**e**) Sc of α7-receptor-mediated nicotinic response. (**f**) Sr of purinergic-mediated response. (**g**) Sr of M_3_-receptor mediated muscarinic response. (**h**) Sr of M_2_-receptor-mediated muscarinic response. (**i**) Sr of α3-receptor mediated nicotinic response. (**j**) Sr of α7-receptor-mediated nicotinic response. *p ≤ 0.05 to respective control group (PBS) values.

Next, we isolated the urinary bladders from CIE mice to measure detrusor muscle tone at the baseline, assess the peak force (PF) of the contractile responses to EFS, high potassium solution and pharmacological activation by different drugs. *In vitro* contractility studies determined a decreased basal tone of the detrusor (by 48.59% to REC group, *p* ≤ 0.05, Fig. 5a) and diminished amplitude of nerve-mediated contractions in the C-RELAP group in response to EFS (by 25.50% to C-PRO group, *p* ≤ 0.05, Fig. 5b). Bladder smooth muscle strips from C-PRO group had a significantly elevated contractile response to EFS (by 174.09% to control, *p* ≤ 0.05, Fig. 5b) and increased PF of muscle-mediated contractions (in response to high potassium, by 199.77% to control, p ≤ 0.05, Fig. 5c). Additionally, percentage of maximal purinergic responses of nerve-mediated contractions normalized to L_0_ were up-regulated in C-PRO group when compared to control group (*p* ≤ 0.05, Fig. 5d) whereas muscarinic responses in C-PRO group were comparable to the control group (Fig. 5e). We further dissected the muscarinic components (Fig. 5f) from nicotinic components (Fig. 5g) of the contractile responses to EFS but neither of them revealed significant differences between experimental groups. To evaluate the velocity of both contractile and relaxing responses after application of EFS, we calculated the contractile and relaxing slopes of purinergic, M3-mediated muscarinic, M2-mediated muscarinic, α-3 nicotinic and α-7 nicotinic responses. The purinergic component of bladder smooth muscle strips contractions from C-PRO mice showed significantly increased slope values of both contraction (Fig. 6a–e) and relaxation phases (Fig. 6f–j). Interestingly, the purinergic, α3- and α7-nicotinic components of the detrusor contractile responses in C-RELAP group of mice showed a significantly delayed relaxation rates as evidenced by the Sr slope (Fig. 6f,i,j).

## Discussion

The long-term follow-up of CIE mice revealed three phenotypes of neurological symptom development which were associated with heterogeneous neurogenic bladder dysfunction. The symptoms of neurodegenerative changes in CIE mice included wobbly gait, hindlimb paresis, quadriparesis and paralysis, as previously reported^21^. The pattern of CSS fluctuations in CIE mice within the entire period of observation (8–10 wks) was used to assign the animals to one of the following experimental groups: group with recovery from initial symptoms after acute phase of the disease (REC group); chronic group with progression of neurodegenerative changes (C-PRO group), and chronic group with relapsing and remitting episodes (C-RELAP group). Each subgroup of CIE mice had unique features regarding bladder function as assessed by *in vitro* and *in vivo* studies.

The types of neurodegenerative symptoms observed in CIE mice closely reflect the features of symptom progression in MS patients. Dysfunctional level of mobility in MS varies depending on the stage and severity of the disease^26^. Based on a recently updated classification of MS phenotypes in humans^27^, C-PRO subgroup of CIE mice may correspond to the clinically “progressive type” of human MS, whereas C-RELAP subgroup is reflective of a “relapsing-remitting phenotype” in MS patients. REC subgroup in CIE mice is characterized by the recovery from the initial neurological impairment and absence of active CSS which corresponds to a type of MS which is not active but could still be progressing^27^. While the development of several distinguished clinical phenotypes in CIE mice provides an additional evidence of the high translational value of the viral murine MS model for future pharmacological and therapeutic studies, further experiments including MRI imaging in CIE mice would be helpful to better correlate the observed clinical symptoms in animals with the MS phenotypes in humans.

Progression of MS symptoms in patients is closely associated with the degree of inflammation in the CNS characterized by expression levels of cytokines and growth factors. The phenotypes of neurological impairment and bladder dysfunction observed in CIE mice correlated with functional modulation of the cytokines and growth factors in the CNS and urinary bladder. For example, C-PRO group showed a significantly increased TGF-β in the bladder as compared to PBS mice, but the same growth factor was unchanged in the other two CIE subtypes, REC and C-RELAP group. TGF-β along with IL-10 are known to be a co-inhibitory molecule expressed in peripheral regulatory immune cell population, which is forkhead box P3 (FoxP3) + regulatory T cells (Tregs) and type 1 regulatory T cells (Tr1). The peripheral regulatory immune cells expressing TGF-β and/or IL-10 are known to trigger autoreactive T cells anergy under normal conditions^28^. We speculate that an up-regulation of TGF-β in the urinary bladder of C-PRO group may reflect TGF-β-mediated inhibitory feedback loop in response to continuous progression of neurological impairment, which is not present in C-RELAP and REC group. On the other side, INF-γ, IL-2 and TNF-α, which are known to trigger progression of MS, are significantly up-regulated in the urinary bladder of C-PRO group^29,30^. Furthermore, in both C-RELAP and C-PRO group, the changes in cytokine content in the brain and urinary bladder suggest a functional cross-talk between the two systems. This speculation is supported by the previous studies which established a correlation between the functional connectivity within brain regions and voiding behavior in MS patients^31^, thereby, providing evidence of the relationship between pyramidal impairment in the lower limb and the severity of LUTS in MS patients^4,7^. The underlying mechanisms of such association are still unclear but could be partly explained by somatic-visceral convergence in the neural control of the lower limb and urinary bladder function^4,32^. Recent studies also suggest the contribution of peripheral nervous system and changes in the visceral organs to the modulation of LUTS in MS patients^33^. This is supported by the reports that local administration of lidocaine and cannabinoids in the urinary bladder of MS patients with neurogenic detrusor overactivity was proven to be therapeutically effective to improve voiding dysfunction^34–37^.

Analyses of bladder function by FPA and cystometry *in vivo*, as well as of detrusor contractility by organ bath studies *in vitro* revealed differential phenotypes of voiding dysfunction between subgroups of CIE mice. Mice in C-RELAP group showed a delayed recovery of voiding function based on the number of large voiding spots. Additionally, *in vitro* contractility studies determined a decreased basal detrusor tone and reduced amplitude of nerve-mediated contractions in C-RELAP group, with purinergic responses of nerve-mediated contractions being up-regulated in C-RELAP group. These data provide evidence that animals with relapsing-remitting type of neurodegenerative changes in the CNS had the most severe type of voiding dysfunction in comparison to other groups. The differences in the types of bladder dysfunction between the CIE mice suggest that this animal model could be used for the testing of novel therapeutic approaches to treat LUTS based on the degree and phenotype of neurological impairments in MS patients^38^. The majority of currently available and widely used oral medications for neurogenic bladder dysfunction include the anti-muscarinic agents^39^. Muscarinic receptors predominately expressed in the urinary bladder are M2 and M3 subtypes known for their role in modulation of bladder reflexes^38^. However, due to observed up-regulation of purinergic responses in C-RELAP group and down-regulation of muscarinic responses in C-PRO group, conventional anti-muscarinic agents may have limited effects in respective groups of MS patients. Although purinergic receptor-mediated responses in the urinary bladder of MS patients^40^ and animals having demyelination^41^ had been previously mentioned, little is known about the underlying mechanisms. One publication showed that purinergic receptor P2X1 expression in the urinary bladder of mice with EAE-driven demyelination was significantly lower than in control mice^42^. Major nicotinic subtypes of receptors in the bladder urothelium are α-3 and α7-isoforms which participate in regulation of bladder excitatory and inhibitory reflexes, respectively. Therefore, significant changes in slope values of relaxation (Sr) of both α3- and α7-mediated responses observed in C-RELAP group may explain why the C-RELAP mice showed irregular and atypical patterns of micturition cycle reflected in Fig. 4.

In conclusion, our study identified several phenotypes of neurogenic bladder dysfunction based on the patterns of neurological impairment development in CIE mice suggestive of high clinical relevance of the viral murine model to the human MS. The mechanistic differences between the subgroups of CIE mice make the model suitable for studying the relationship between voiding dysfunction and progressive neurological impairment over the course of virus-induced demyelinating disorder, and to test novel therapeutic approaches for the treatment of human MS.

## Materials and Methods

### Study approval

The study protocol was approved by the Institutional Animal Care and Use Committee (IACUC) of the University of Colorado Denver. All experiments were performed in accordance with relevant guidelines and regulations.

### Murine CIE model

Adult male mice (C57BL/6J, 8 weeks of age, Jackson Laboratories) received a single intracranial inoculation of 20 µl PBS (N = 25) or mouse hepatitis virus (MHV, A59 strain, 5,000 PFU, N = 72) in 20 µl PBS under isoflurane anesthesia. MHV was prepared as previously described^21,43^. After inoculation, mice were observed daily for evaluation of neurological symptom development by using clinical symptoms score (CSS) system and weight every week up to 10 wks post-inoculation. The clinical symptom score (CSS) was assigned based on the following scale: 0 = normal with no clinical signs, 1 = loss of tail tonicity/kyphosis, 2 = tail paralysis/severe kyphosis, 3 = partial hindlimb paralysis, 4 = complete hindlimb paralysis, and 5 = complete hindlimb paralysis and forelimb paresis/paralysis as previously described^21^. Animals were weighed both before inoculation (baseline) and every subsequent week until the experimental end point. All mice had *ad libitum* access to food and water with special measures taken to accommodate neurologically compromised animals.

### Criteria for subgroup assignment in CIE mice based on the profile of neurodegenerative symptoms

After inoculation with the virus, animals were observed daily up to 10 weeks to evaluate the degree and time-dependence of neurological symptom development graded by the CSS. After instillation of MHV, the degree of neurologic impairment was evaluated daily by CSS, as previously described^21^. Following the CSS fluctuations, we decided to group the animals based on the severity, duration and persistence/recovery of neurological symptoms determined by CSS (Fig. 1a). The most interesting group of animals was the one with occurring relapsing and remission episodes (C-RELAP group), which mimicked the most prevalent phenotype of MS in humans^44^ characterized by the periods of symptom worsening (relapses or flare-ups) followed by the periods of recovery from the symptoms between relapses^27^. The animals were assigned to the C-RELAP group if they met all of the following criteria: 1 – the presence of a symptom-free period for at least 24 hours (hrs) after recovery from the initial rise in CSS; 2- the presence of at least 2 symptom-free periods (24 hrs duration each), and 3 - CSS > 2 during the period of relapse (re-occurrence of symptoms after a remission episode). Animals were assigned to recovery group (REC) and chronic progression of symptoms (C-PRO group) based on the absence or presence of symptoms at experimental end point with no observed relapsing-remitting episodes. The algorithm followed to assign a mouse to one of the described neurological groups is presented in Fig. 1a.

### Expression profile of cytokines and growth factors determined by multiplex ELISA assay

The total protein was extracted from frozen brain, spinal cord and urinary bladder tissues using radioimmunoprecipitation (RIPA) buffer containing protease and phosphatase inhibitors cocktail. The tissues were isolated at the experimental end point (10 weeks post-inoculation) from the animals assigned to the cystometry group. Multiplex ELISA was performed (Quantibody Custom Array Kit, QAA-CUST, Raybiotech, Norcross, GA) in accordance with manufacturer’s instructions. On the 12-plex panel of INF-γ, IL-1β, IL-2, IL-4, IL-5, IL-6, IL-10, IL-12, IL-17, TGF-β and TNF-α, 400 μg of protein was loaded and incubated overnight at 4 °C. After washing with Wash Buffer I & II, 80 μl of detection antibody cocktail was added to each well and incubated at room temperature (RT) for 2 hours. After washing, 80 µl of anti-streptavidin secondary antibody (800CW, LiCor, Lincoln, NE) was added and incubated for 2 hours at RT. The samples were decanted and washed in Wash Buffer I five times for 5 minutes at RT.

### Micturition patterns evaluated by filter paper assay

To follow micturition patterns *in vivo*, animals were subjected to filter paper assay (FPA) once a week as previously described^45^. Animals were placed in individual cages containing elevated (by 1 inch) mesh flooring for 3 h with filter paper (Filter Paper Qualitative Advantec, Tokyo Roshi Kaisha Ltd, Japan) being on the bottom of the cage under the mesh. Each mouse had free access to sterile water gel (HydroGel, Clear H2O, Westbrook, ME) with no food provided during the testing. Every animal underwent FPA evaluation at the following time points: pre-inoculation (baseline) and every week up to 8 wks. The collected filter papers were imaged under UV light by Labnet DyNA UV Transilluminator (Labnet International, Inc., Edison, NJ), DigiDoc-It Imaging System (UVP, Cambridge, UK) and DigiDoc-It Imaging Software (UVP, Cambridge, UK). The number of large (≥1.0 cm) and small (<1.0 cm) micturition spots were summed after each test for each mouse.

### Urodynamic evaluation of bladder function in freely moving mice

Animals assigned for urodynamic evaluation of urinary bladder function (awake cystometry) underwent a survival surgical procedure to insert bladder catheters as previously described^46^. Briefly, the animals were anesthetized with isofluorane (VEDCO, St. Albans, VT) and the bladder was exposed through a lower midline abdominal incision. A polyethylene tubing (PE-50, I.D. 0.58 mm, O.D. 0.96 mm; Intramedic, Becton Dickinson. Parsippany, NY) with a flared end was inserted through a puncture at the bladder dome, and sutured in place with purse string suture and 7.0 silk (Ethicon, Somerville, NJ). The catheter was then tunneled subcutaneously and exteriorized at the scapular region where it was sutured to the skin and filled with sterile saline solution. After confirming no leakage in the bladder, the catheter was plugged with a metal rod, and the muscle and skin layers were closed with a 5.0 silk suture (Ethicon, Somerville, NJ). Particular care was taken not to stretch the bladder during the procedure or to restrict the normal bladder movement once the catheter was in place.

Mice were given 4–5 days to recover from surgery before the initiation of cystometric evaluation of bladder function. Conscious mice were placed in the cystometry cages (length: 24 cm, width: 16 cm, and height: 12 cm) without restraint and allowed to acclimate for 30 min. The tip of the exteriorized bladder catheter, located at the base of the mouse neck, was connected to a pressure transducer and the infusion pump of the cystometry station (Small Animal Laboratory Cystometry, Catamount Research, and Development, St. Albans, VT) using a T-shaped valve. Room temperature saline solution (0.9% NaCl) was infused into the bladder at a rate of 10 μl/min. Voided urine was collected in a tray connected to a force displacement transducer, which integrated the results into the data-acquisition program. Each animal was observed for approximately six to eight voiding cycles. The urodynamic parameters were continuously recorded using the MED-CMG Small Animal Cystometry acquisition software and analyzed with the Cystometry Data Analysis software v 1.4 (Catamount Research and Development). The following cystometric parameters were evaluated: bladder capacity, pressure at the start of micturition, micturition rate, intravesical pressure, inter-micturition interval (presented as the ratio of the cycle duration in each of the experimental groups to the control group), and number of non-voiding contractions. Non-voiding contractions were defined as the increased values in detrusor pressure not associated with voiding, equal or larger than twice the mean value of baseline. Normal voiding contractions had amplitudes of at least a third of maximal pressure during a single micturition event.

### Contractility and relaxation of the detrusor muscle *in vitro*

Bladder smooth muscle (BSM) strips were isolated from control and CIE mice following the previously described procedures^25,47^. Briefly, murine urinary bladders were cut in half and placed in individual organ baths (Radnoti LLC, Monrovia, CA, USA), containing 7 ml of normal Tyrode Buffer (TB, in mM: NaCl 130.0, KCl 5.0, CaCl_2_ 1.7, MgCl_2_ 1.0, NaH_2_PO_4_ 1.3, NaHCO_3_ 17.0, Glucose, 10.0. pH 7.4) maintained at 37 °C and equilibrated with a constant supply of 95% O_2_–5% CO_2_. One end of each bladder strip was attached to a glass rod at the bottom of the organ chamber. The other end was attached to a force displacement transducer connected to a computerized system for data acquisition and analysis (AD Instruments, Colorado Springs, CO, USA). After a 1 h equilibration, the length of optimal force development (*L*_o_) was determined by manually increasing the length of each strip by ~1.5 mm increments until the maximal contractile force in response to electric field stimulation (EFS; 80 V, 32 Hz, 1 ms) was achieved. The bath solution was then changed to fresh TB containing the drugs to isolate cholinergic and purinergic responses after EFS stimulation. Intact bladder stips were used for each pharmacological evaluation to avoid desensitivation of cholinergic and purinergic receptors during the wash-off process between the drugs. The following drug combinations were used: 1 µM of atropine (non-selective mAChR antagonist, Sigma, St. Louis, MO) + 300 nM of TMPH (nicotinic antagonist, Tocris Bioscience, Bristol, United Kingdom) were used to isolate the purinergic component of the contractile response; 30 µM of α,β-Methylene ATP (purinergic receptor desensitizer^25^, Tocris Bioscience, Bristol, United Kingdom) was used to isolate the cholinergic component; 30 µM of α,β-Methylene ATP + 300 nM of TMPH (Tocris Bioscience, Bristol, United Kingdom) were used to isolate the muscarinic component of contractions; 30 µM of α,β-Methylene ATP + 300 nM of TMPH + 1 µM of Methoctramine (M2 mAChR antagonist, Sigma, St. Louis, MO) were used to isolate the M3 muscarinic component; 30 µM of α,β-Methylene ATP + 300 nM of TMPH + 10 µM of darifenacin (M3 mAChR antagonist, Sigma, St. Louis, MO) were used to isolate M2 muscarinic component of contractions; 1 µM of atropine + 30 µM of α,β-Methylene ATP were used to isolate the nicotinic component; 1 µM of atropine + 30 µM of α,β-Methylene ATP + 1.4 nM of Methyllycaconitine Citrate (antagonist of α-7 nicotinic receptors, Tocis Bioscience, Bristol, United Kingdom) were used to isolate the α3-mediated nicotinic component of contractions; 1 µM of atropine + 30 µM of α,β-Methylene ATP + 440 nM of TC 1698 (agonist of α7 nicotinic receptors, Tocris Bioscience, Bristol, United Kingdom) were used to isolate the α7-mediated nicotinic component of contractions. After incubation of the bladder strips with these drugs for 15–20 minutes, the BSM were stimulated with 5, 10, 15, 20, 25 and 30 Hz of EFS to evaluate the contractile responses. Some of the intact strips also underwent a KCl test (bath solution was replaced with TB containing 125 mM KCl) to evaluate muscle-mediated contractility of the detrusor in different experimental groups of animals. All BSM experiments were recorded and analyzed using LabChart 8 Pro (AD Instruments, Colorado Springs, CO, USA), pClamp 10 (Molecular Devices, LLC. San Jose CA, USA) and Matlab R2014b (MathWorks, Natick, MA, USA). The following parameters were measured and analyzed: basal muscle tone (in g/g, g of force per g of muscle strip weight), peak force (PF; in g or g/g when normalized to the weight of the muscle strip), integral force (IF) and maximal slope of contraction (Sc) or relaxation (Sr) phases as previously described^38^.

### Statistics

All data are presented as mean ± SE. Results were statistically analyzed using paired t-test to compare the parameters from the same group at different time points, and unpaired t-test to compare the parameters between different groups (Systat Software, Inc. San Jose, CA). Data with *p* ≤ 0.05 difference were considered statistically significant.

## Data Availability

The data associated with this manuscript is available.
